# Multifaceted role of T-box transcription factor 4: From embryonic development to disease pathogenesis

**DOI:** 10.1016/j.gendis.2025.101811

**Published:** 2025-08-14

**Authors:** Lin Yi, Li Zhou, Bianfei Shao, Tingxiu Xiang, Jingyi Tang

**Affiliations:** a Chongqing Key Laboratory of Translational Research for Cancer Metastasis and Individualized Treatment, Chongqing University Cancer Hospital, Chongqing 400030, China; b School of Pharmacy and Bioengineering, Chongqing University of Technology, Chongqing 400054, China; c The First College of Clinical Medicine, Chongqing Medical University, Chongqing 400016, China

**Keywords:** Cancers, DNA methylation, Embryonic development, TBX family, TBX4

## Abstract

T-box transcription factor 4 (TBX4), a crucial member of the T-box gene family, is essential for embryonic development, particularly in the formation of hindlimbs and lungs. Beyond these developmental roles, TBX4 is integral for maintaining the structural integrity and function of the respiratory, motor, and nervous systems. Dysregulation of TBX4 is implicated in serious diseases, including pulmonary hypertension, small patella syndrome, and tracheal stenosis, with mutations and aberrant expression patterns emerging as potential diagnostic markers. Additionally, TBX4 contributes to tumorigenesis in cancers such as pancreatic, lung, and bladder cancers, where recent studies suggest DNA methylation as a primary mechanism underlying TBX4 suppression, positioning it as a promising prognostic marker. Despite these advances, the precise functions and regulatory mechanisms of TBX4 remain insufficiently understood. This review consolidates current knowledge on the roles and molecular mechanisms of TBX4 in mammalian embryonic development and its association with diseases, highlighting the need for further research into its contributions to human health.

## Background

T-box transcription factor 4 (*TBX4*) is a key member of the T-box gene family, which includes genes such as *TBX1*, *TBX2*, *TBX3*, *TBX4*, and *TBX5*. These genes are characterized by the T-box domain, essential for DNA binding and transcriptional regulation.^1^^,^^2^ It also plays key roles in embryonic development and the formation of various organs (such as the heart, hindlimbs, lungs, and breasts),^3^, ^4^, ^5^, ^6^, ^7^ and is associated with multiple diseases. For example, *TBX1* dysregulation is linked to 22q11.2 deletion syndrome,^8^ *TBX3* to ulnar-mammary syndrome,^9^ *TBX4* to stiff-person syndrome (SPS)^10^ and developmental dysplasia of the hip (DDH),^11^ and *TBX5* to Holt-Oram syndrome.^12^ Aberrant expression or mutations in these genes often lead to specific developmental defects and diseases. Among them, the *TBX4* gene is particularly fascinating and warrants focused investigation due to its significant role in limb development and associated diseases. *TBX4* plays a critical role in embryonic development, particularly in the formation of the hindlimbs and lungs. Since its initial discovery in mice in 1996, the central role of *TBX4* in embryonic patterning and morphogenesis has been progressively elucidated.^13^ Subsequent studies confirmed the presence of *TBX4* in the human genome and further clarified its role in axial limb patterning.^14^

Over the years, accumulating evidence has revealed that *TBX4* is indispensable not only for normal development but also for maintaining tissue homeostasis, with dysregulation contributing to a variety of human diseases.

In the musculoskeletal system, mutations in *TBX4* are causally linked to SPS, and its haploinsufficiency has been identified in several familial cases.^15^ Additionally, *TBX4* has been implicated in DDH and congenital clubfoot, reinforcing its importance in skeletal development and joint formation.^11^

In the respiratory system, mutations or aberrant expression of *TBX4* are strongly associated with pulmonary arterial hypertension (PAH)^16^^,^^17^ and tracheal stenosis^18^ in several cases, particularly in pediatric populations. These associations highlight TBX4’s critical role in normal pulmonary vascular, lung branching morphogenesis,^19^ and tracheal and bronchial cartilage development.

More recently, *TBX4* has garnered increasing attention in the field of oncology. It is emerging as a potential tumor suppressor,^20^ with studies reporting significant correlations between reduced TBX4 expression and advanced tumor grade, poor prognosis, and decreased overall survival in several cancer types.^21^ These discoveries have positioned *TBX4* as a critical molecular player in developmental biology and disease pathogenesis, thereby making it a prominent focus in both basic and translational research.

## The structure and function of *TBX4*

*TBX4* is located on chromosome 17q23.2^22^ and belongs to the T-box family of transcription factors. It features a highly conserved T-box domain, approximately 180 amino acids in length, essential for DNA binding and transcriptional regulation.^23^ This domain comprises several functional regions: the T-box binding element (TBE), the N-terminal domain, the transactivation domain, and the C-terminal domain. The TBE contains highly conserved DNA-binding sequences critical for its function.^24^^,^^25^ The N-terminal domain includes a basic amino acid sequence essential for DNA interaction.^25^ The C-terminal subdomain may regulate protein stability and nuclear localization.^26^^,^^27^ Importantly, regions outside the T-box domain exhibit distinct interaction potentials compared with other protein effectors.^13^

While *TBX4* shares structural similarities with other T-box transcription factors, it possesses unique sequence characteristics that may contribute to its specific regulatory roles in embryonic development and tissue differentiation. The T-box, a conserved DNA-binding region within this family, has remained largely unchanged through evolutionary history, underscoring its fundamental role in DNA interaction. However, the protein regions outside of the T-box harbor distinct potential interactions, suggesting TBX4’s unique regulatory capabilities.^13^ For example, *TBX4* not only regulates protein-coding genes but may also influence the expression of non-coding RNAs, particularly long non-coding RNAs (lncRNAs).^28^

**Figure 1:**
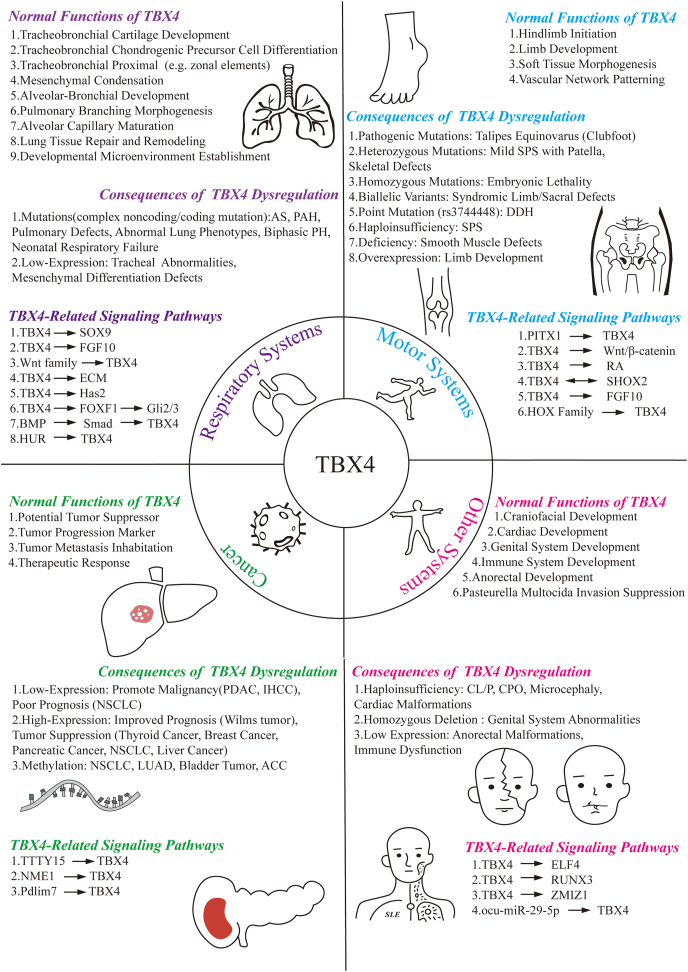
Functional network and cross-system regulatory mechanisms of TBX4. This figure illustrates the multifaceted regulatory roles of TBX4 and its pathological associations across the respiratory, musculoskeletal, and other organ systems, as well as in tumorigenesis. In the respiratory system, TBX4 governs tracheobronchial cartilage formation, alveolar and bronchiolar branching morphogenesis, and lung tissue repair. Mutations or reduced expression are linked to pulmonary arterial hypertension (PAH), neonatal respiratory failure, and tracheal malformations. Within the musculoskeletal system, TBX4 regulates hindlimb initiation, soft tissue morphogenesis, and vascular network patterning, while heterozygous mutations or point mutations (*e.g.*, rs3744448) are linked to stiff-person syndrome (SPS) and developmental dysplasia of the hip (DDH). In other systems, TBX4 contributes to craniofacial, cardiac, genital, and anorectal development, and modulates immune homeostasis via regulation of ELF4, RUNX3, and ZMIZ1. Dysregulation of TBX4 is implicated in conditions such as cleft lip/palate, isolated cleft palate, and genital system malformations. In cancer, TBX4 exhibits dual regulatory roles: low expression drives malignant progression in non-small cell lung cancer and pancreatic ductal adenocarcinoma, while high expression exerts tumor-suppressive effects in Wilms tumor and thyroid cancer, potentially mediated through epigenetic modifications (*e.g.*, methylation) and lncRNA regulation (*e.g.*, TTTY15/NME1 axis). At the molecular level, TBX4 orchestrates organ morphogenesis via the SOX9–FGF10–BMP pathway, mediates tissue repair through Wnt/β-catenin and RA signaling, and dynamically modulates the tumor microenvironment via non-coding RNAs (*e.g.*, ocu-miR-29-5p). This integrative framework highlights TBX4’s complex regulatory network, providing a systematic perspective on its roles in development, immunity, and disease pathogenesis.

*TBX4* is essential for both embryonic development and tissue regeneration. It plays a pivotal role in limb development^29^^,^^30^ and contributes to the formation of the respiratory^31^ and the urinary system.^13^ Specifically, *TBX4* influences the development of both skeletal and soft tissues in the foot^32^^,^^33^ and is critical for the normal formation of the trachea, bronchi, and lungs.^31^ Furthermore, TBX4 is implicated in tissue regeneration processes, facilitating repair and regeneration.^34^ Collectively, TBX4 serves multiple functions in proliferation, differentiation, and maintaining tissue integrity, crucial for preserving the structural and functional aspects of both embryonic and adult tissues. Dysregulation or mutations in TBX4 can lead to developmental defects and contribute to severe diseases. Here, we summarize the functional network and cross-system regulatory mechanisms of TBX4 in Figure 1. In this review, we further elaborate on the diverse functions of TBX4 within different physiological systems.

## TBX4’s role in the respiratory system and its associated mechanisms

### Respiratory system development

Initially, the critical role of TBX4 gene in tracheal development was validated in mammals. Studies show that *Tbx4* is a key factor controlling the development of tracheal and bronchial cartilage,^31^ differentiation of chondrocyte precursor cells,^35^ and formation of proximal structures like tracheal rings.^36^ Mechanistically, *Tbx4* modulates extracellular matrix components to promote mesenchymal condensation, thereby regulating the expression of Sry-box transcription factor 9 (Sox9).^31^ *Sox9* is essential for chondrogenesis, and reduced expression of its downstream genes *Sox5* and *Sox6* results in abnormal tracheal cartilage development. Furthermore, *Tbx4* acts as an upstream regulator of *Sox9* and can partially compensate for Sox9 loss-of-function, underscoring its essential role in tracheal morphogenesis.^31^^,^^37^ Cebra-Thomas et al (2003) found that inhibiting Tbx4 and Tbx5 expression reduced fibroblast growth factor 10 (FGF10) levels in mesenchyme, thereby impairing lung branching and indirectly disrupting normal tracheal development.^38^

TBX4’s regulation in the respiratory system heavily relies on the Wnt signaling pathway. The Wnt family consists of 19 secreted glycoproteins that control various developmental processes, including cell fate determination, proliferation, polarity, and migration.^39^ During early embryonic development, Wnt2 and Wnt2b activate the Wnt/β-catenin pathway, significantly influencing tracheal formation.^40^ In tracheal development, specific Wnt ligands (such as Wnt7b, Wnt5a, and Wnt2/2b) are crucial for TBX4 expression and tracheal mesoderm differentiation, as they regulate differentiation of tracheal mesodermal cells and thus affect normal tracheal formation.^13^^,^^41^^,^^42^ Research indicates that RNA-binding protein HuR plays a key role in regulating mesenchymal responses during lung branching, potentially indirectly affecting the Wnt/β-catenin signaling pathway.^43^^,^^44^ HuR deficiency reduces the stability of FGF10 and Tbx4 mRNAs, leading to decreased expression of corresponding proteins in lung mesenchyme, which adversely affects tracheal development and may cause mesenchymal defects.^43^

### Apert syndrome

In humans, the *TBX4* gene has been implicated in the pathogenesis of Apert syndrome, a rare form of acrocephalosyndactyly characterized by craniofacial abnormalities, limb deformities, and intellectual disability.^45^^,^^46^ In 2009, Tiozzo et al demonstrated a correlation between tracheal stenosis and increased mesenchymal cell proliferation, positively associated with elevated expression of Fgf10 and its upstream regulator, Tbx4.^18^ In the same year, Suhrie et al further strengthened the association between *TBX4* gene mutations/deletions and Apert syndrome-related symptoms, particularly neonatal respiratory failure, highlighting the critical role of *TBX4* in respiratory system development and function.^47^^,^^48^

**Figure 2:**
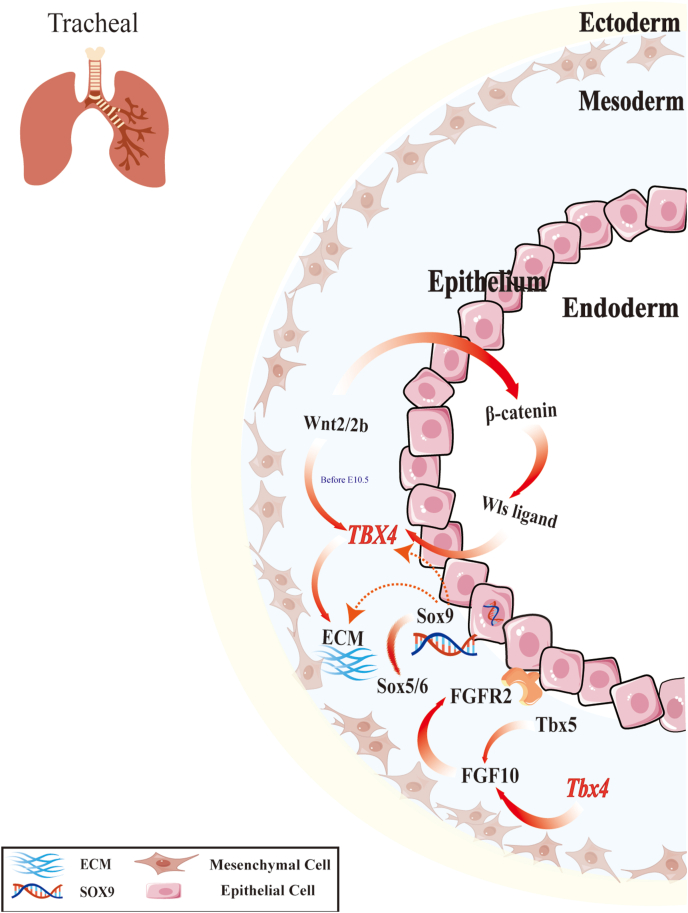
Integrated regulatory network of TBX4 in tracheal development. This figure illustrates that *TBX4* serves as a pivotal regulator in tracheal formation through the coordinated interplay between mesodermal and endodermal tissues. It drives the expression of FGF10, vital for determining tracheal morphology and function, while FGF10 signaling cascades enhance bronchial development. TBX4 stimulates tracheal cartilage formation by up-regulating *Sox9* and promoting mesenchymal condensation, with downstream *Sox5* and *Sox6* implicated in cartilage development. *TBX4* may directly regulate *Sox9* or indirectly via extracellular matrix condensation modulation. Wnt signaling also plays a role, with Wnt ligands activating β-catenin to govern tracheal epithelial cell differentiation. Wnt2/2b is particularly active before E10.5, shaping the tracheal epithelium. Wnt signaling regulates Tbx4 expression, which in turn controls tracheal morphogenesis. The extracellular matrix provides structural support, and TBX4’s modulation of it indirectly impacts Sox9 expression and cartilage formation. These interconnected mechanisms ensure proper tracheal development and function.

To visualize the intricate molecular interactions involving TBX4 in tracheal development, please refer to Figure 2, which illustrates the signaling pathways and cellular responses that are regulated by TBX4 and associated factors.

### Lung tissue repair

After lung injury, increased activity of the extracellular signal-regulated kinase (ERK)/mitogen-activated protein kinase (MAPK) signaling pathway suppresses Tbx4 expression, thereby affecting lung tissue repair and remodeling.^49^^,^^50^ Meanwhile, a positive feedback loop exists between Tbx4 and phosphorylated Smad1/5, reinforcing its role in bone morphogenetic protein (BMP)-mediated signaling.^51^ During lung development, the BMP signaling pathway mediates alveolar and bronchial formation.^52^ Under pathological conditions, reduced BMP-Smad1/5/8 signaling coupled with increased transforming growth factor β (TGFβ)-Smad family member 2/3 (SMAD2/3) signaling is believed to be associated with repair of lung injury.^53^, ^54^, ^55^

### Pulmonary arterial hypertension

In humans, *TBX4* is also related to lung developmental diseases, particularly PAH. PAH is characterized by elevated blood pressure in lung arteries, leading to symptoms such as shortness of breath, fainting, fatigue, chest pain, leg swelling, and rapid heartbeat.^56^^,^^57^ Studies have found significant associations between *TBX4* gene mutations and PAH occurrence, observed in both pediatric and adult cases. Over a decade ago, a Dutch clinical study first pointed out that *TBX4* gene mutations increased individual susceptibility to PAH, highlighting the important role of *TBX4* in pulmonary vascular function.^16^ Further research revealed that complex noncoding variants in TBX4 enhancer regions synergized with TBX4 coding mutations to exacerbate lung defects, leading to severe lung abnormalities and PAH.^58^, ^59^, ^60^

Particularly noteworthy is that *TBX4* gene mutations have more pronounced effects in pediatric PAH patients, with a higher incidence in children than adults.^61^^,^^62^ For example, variant sites, such as rs3744439, are associated with increased PAH susceptibility, suggesting that specific TBX4 loci may influence individual risk profiles.^63^ In 2019, Karolak et al proposed that abnormal lung phenotypes caused by TBX4 variants may stem from polygenic interactions,^64^ implying that TBX4 is part of a complex genetic network involved in lung pathophysiology. Additionally, Galambos et al reported associations between *TBX4* gene mutations, 17q23 deletions, and severe biphasic pulmonary hypertension, with these patients often also exhibiting skeletal and cardiac abnormalities.^60^ Tsoi et al further demonstrated that *TBX4* may be one of the main factors leading to persistent PAH in patients.^65^ Rosenbaum et al observed different expression patterns of the *TBX4* gene during PAH progression, with this variability potentially influencing disease progression and clinical outcomes.^66^ In 2020, van den Heuvel et al noted that identifying likely pathogenic/pathogenic variants on the *TBX4* gene in patients could help screen high-risk relatives, enabling early detection of PAH.^67^ Moreover, patients with TBX4 syndrome exhibit a range of symptoms, including perinatal cardiopulmonary disease, SPS, and PAH.^68^

### Other lung diseases

TBX4’s role in the lung developmental microenvironment is also associated with other lung diseases. TBX4 helps regulate the extracellular matrix, particularly by influencing the chondroitin sulfate proteoglycan versican in the amnion.^69^ This finding suggests that versican may be a key extracellular matrix target regulated by *TBX4* during lung development. Lung fibroblasts are the main cellular components of connective tissue, synthesizing and remodeling extracellular matrix components. Under precise regulation by *TBX4*, fibroblasts enhance cellular invasiveness and trigger hyaluronic acid production, more specifically through activation of hyaluronan synthase 2 (Has2) gene expression, thereby creating the necessary microenvironment to support lung development.^34^ Hinck (2004) emphasized the importance of axon guidance proteins, signaling molecules, and catecholamines in shaping the lung microenvironment and promoting healthy lung development.^70^ Recent work by Karolak et al using chromatin immunoprecipitation sequencing technology revealed associations between *TBX4* and binding sites in axon guidance pathways, which are crucial for lung bud formation.^28^

Recent studies have also examined the importance of *TBX4* in regulating gene expression and function from the perspective of microRNAs, particularly in the context of lung development and disease. Three microRNAs, namely, miR-102, miR-301c, and miR-589, have been shown to regulate Tbx4 expression and function through different mechanisms, significantly impacting lung development and related pathologies. miR-102 and miR-301c reduce Tbx4 expression by binding to Tbx4 mRNA, promoting its degradation or inhibiting translation. In contrast, miR-589 binds to Tbx4 mRNA to form stable complexes that promote its translation, thereby increasing Tbx4 protein levels.^71^

### Lung system development in other species

In murine and avian models, *Tbx4* has also been shown to govern lung development. It regulates lung branching morphogenesis by modulating *Fgf10*, a key growth factor essential for alveolar formation, airway branching, and epithelial–mesenchymal interactions.^31^^,^^36^^,^^64^^,^^72^, ^73^, ^74^ Through the *Tbx4–Fgf10* signaling axis, TBX4 ensures the proper structural development of the alveoli and bronchi.^31^^,^^64^^,^^72^ For example, Cebra-Thomas et al (2003) found that inhibiting Tbx4 and Tbx5 expression reduced Fgf10 levels in mesenchyme, thereby suppressing lung branching.^38^ Additionally, *Fgf10* binding to its receptor *Fgfr2* actively regulates the Sonic hedgehog (Shh) signaling pathway during lung development.^75^^,^^76^ In the absence of Shh epithelial-mesenchymal signaling, elevated GLI family zinc finger 3 repressor (Gli3R) levels may adversely affect Tbx4 expression and lead to defects in proliferation and differentiation of lung mesenchymal cells.^77^ Both *Tbx4* and *Fgf10* are regulated by the Shh signaling pathway, a process potentially related to lethal lung developmental disorders and alveolar capillary dysplasia with misalignment of pulmonary veins. Ets variant transcription factors (ETV4 and ETV5) participate in regulating the FGF-Shh feedback loop,^78^ while forkhead box F1 (Foxf1) is also influenced by Shh signaling.^78^ *Tbx4* may affect lung branching morphogenesis by regulating Foxf1 expression^31^^,^^52^^,^^78^.

In conclusion, TBX4 plays a central role in respiratory system development and function through its regulation of key signaling pathways, including FGF10, Wnt, and BMP. These interactions are essential for tracheal morphogenesis, lung branching, and alveolar formation. Dysregulation of TBX4 is linked to severe human conditions, such as Apert syndrome, PAH, and lung developmental disorders. Additionally, TBX4’s involvement in extracellular matrix remodeling and microRNA-mediated gene expression further underscores its multifaceted role in lung homeostasis and repair. Insights into TBX4’s mechanisms not only enhance our understanding of respiratory development but also pave the way for potential therapeutic strategies for related diseases.

**Figure 3:**
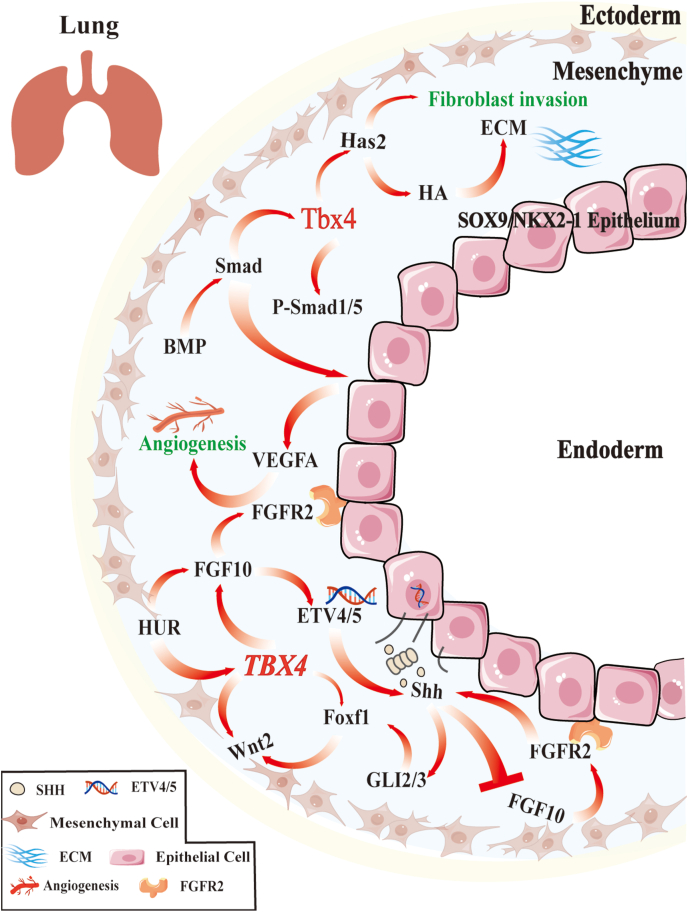
TBX4 coordinates multiscale regulatory networks in lung development. This figure illustrates that TBX4 is pivotal in lung branching morphogenesis, with its expression spanning various cell types, including fibroblasts, perivascular cells, and airway smooth muscle cells, across different lung development stages. Sox9/Nkx2-1 epithelial progenitor cells secrete Shh, which activates Gli2/3 in mesenchymal cells and induces Foxf1 expression. Foxf1 synergizes with *TBX4* to activate Wnt2/2B and *FGF10*, crucial for lung duct formation and growth. Mesenchymal FGF10 activates Fgfr2 in Sox9/Nkx2-1 epithelial progenitor cells, boosting VEGFA production for lung vascularization. The FGF10-Shh feedback loop, mediated by ETV4/5, ensures orderly lung branching. RNA-binding protein HuR may stabilize *FGF10* and TBX4 mRNA, while BMP-Smad signaling modulates Tbx4 expression/function, activating P-Smad1/5 to enhance fibroblast invasiveness. TBX4 also regulates the extracellular matrix by activating Has2, increasing HA to support lung development. These interconnected regulatory mechanisms ensure proper lung branching morphogenesis.

To further explore the complex signaling network that TBX4 is part of in lung development and its implications in pulmonary diseases, Figure 3 provides a detailed illustration of the interactions between TBX4, FGF10, and other signaling molecules.

## TBX4’s role in the motor system and its associated mechanisms

### Hindlimb development

*Tbx4*, together with paired-like homeodomain transcription factor 1 (*Pitx1*), serves as a critical regulator in hindlimb development in mice. *Pitx1* directly modulates Tbx4 expression, thereby establishing hindlimb-specific morphological features. Studies have demonstrated that Pitx1 deficiency reduces Tbx4 levels,^29^^,^^30^ while its aberrant overexpression induces Tbx4 and other hindlimb-specific genes, such as homeobox C10 (Hoxc10) and Hoxc11.^79^^,^^80^ Although Pitx1 remains the only confirmed direct upstream regulator of Tbx4 so far, other regulators may also influence its activity.^79^^,^^81^ Genetically, *Tbx4* initiates hindlimb outgrowth, whereas Pitx1 specifies hindlimb identity, indicating a cooperative role in limb morphogenesis.^35^^,^^80^^,^^82^ Therefore, mutations in Pitx1-binding sites within hindlimb enhancer A (HLEA) disrupt Pitx1 binding, reducing hindlimb Tbx4 expression,^83^ leading to characteristic changes in hindlimb skeletal size but not forelimb skeletal size during development.

In terms of the downstream pathways of *TBX4* in motor system development, here we mainly discuss the Fgf family, Wnt pathway, and retinoic acid (RA) signaling. During early limb development, *TBX4* activates FGF10 expression in the lateral plate mesoderm, which subsequently stimulates Wnt signaling to initiate apical ectodermal ridge (AER) formation. The AER, in turn, expresses Fgf8, forming a positive feedback loop that sustains Fgf10 expression and drives limb bud elongation.^82^^,^^84^, ^85^, ^86^

### Muscle formation

*Tbx4* also contributes to the development of muscle connective tissue by regulating downstream effectors, such as N-cadherin and β-catenin, both of which coordinate with the Wnt signaling pathway to orchestrate soft tissue morphogenesis.^32^^,^^33^ Additionally, *Tbx4* correlates with Fgf10 and Fgf8 expression, inducing Fgf8 in anterior spinal cells and activating Wnt signaling via mesodermal Fgf10.^87^ Tbx4–Wnt interactions also involve allantois-derived angiogenesis, suggesting a role in coordinating limb vascular and tissue development.^31^ In Fgf10^−/−^ embryos, Tbx4 expression is markedly suppressed in prospective hindlimb mesoderm, indicating partial dependence on Fgf10-induced AER formation.^36^ *Tbx4* also collaborates with *Pitx1* to co-regulate Fgf10 expression and downstream signaling cascades.^35^^,^^88^, ^89^, ^90^

In addition to Wnt and Fgf signaling, RA signaling interacts with *Tbx4* to establish a coherent Fgf feedback loop between limb mesenchyme and ectoderm, modulating Fgf10 expression. However, Tbx4 expression may sometimes operate independently of RA signaling.^91^, ^92^, ^93^, ^94^ *Shox2* interacts with *Tbx4* during limb development, suppressing Tbx4 expression (especially in forelimbs), while Tbx4 reciprocally regulates Shox2 transcription and protein levels as a feedback modulator.^95^ A 2012 study also showed that Tbx4 deletion caused abnormal smooth muscle formation.^31^

### Limb disorders

Clinically, *TBX4* has been implicated in various human limb disorders. Here, we mainly discussed SPS, DDH, and clubfoot, which have been confirmed to be the most relevant to *TBX4*.

SPS is a hereditary skeletal dysplasia characterized by patellar hypoplasia or aplasia, along with abnormalities in the pelvis and feet. Typical manifestations include widened interdigital gaps between the first and second toes, altered metatarsal spacing, shortened fourth and fifth toes, and occasional flat feet. Recurrent patellar dislocation is a frequent complication in pediatric and adolescent patients with SPS, and SPS pathogenesis involves *TBX4* mutations or aberrant expression.^10^ Haploinsufficiency is often implicated in familial cases. Heterozygous loss-of-function mutations generally lead to milder phenotypes, whereas homozygous or biallelic mutations are linked to more severe syndromes, such as sacral agenesis and limb deformities, and may even result in embryonic or neonatal lethality.

DDH involves hip socket dysplasia and femoral head malposition during embryogenesis/infancy. As a key hindlimb regulator, *TBX4* contributes to DDH pathogenesis. Specific SNPs (*e.g.*, rs3744448) significantly associate with DDH, particularly in Chinese males, influencing severity in complete dislocations but less so in subluxations or joint instability.^11^

Clubfoot, a congenital deformity marked by varus, equinus, adductus, and cavus positioning of the foot, often involves underlying soft tissue abnormalities. Gurnett et al identified *TBX4* as a direct *PITX1* target, suggesting PITX1–TBX4 axis mutations may promote clubfoot.^96^ However, Lu et al found that *TBX4* variants were not common causes,^97^ implying complex genetic mechanisms. Dobbs et al (2017) proposed PITX1–TBX4–Hoxc pathway mutations may underlie familial clubfoot.^98^ Mutations in the PITX1–TBX4–Hoxc pathway were proposed in familial clubfoot cases,^99^ and additional evidence implicated homeobox genes, including SHOX family—both of which were involved in limb and skeletal development and were associated with short stature phenotypes—in contributing to limb anomalies through interactions with *TBX4*.

### Motor system development in other species

Studies of *Tbx4* in motor system development across various species models have further enriched our understanding of its molecular regulatory mechanisms. *Tbx4* promotes limb outgrowth by activating *Fgf10* expression in the lateral plate mesoderm, which in turn stimulates Wnt signaling and initiates the formation of the AER. The AER expresses *Fgf8*, thereby maintaining *Fgf10* expression in the mesoderm and establishing a positive feedback loop essential for sustained limb bud elongation.^82^^,^^84^, ^85^, ^86^ *Tbx4* also coordinates soft tissue morphogenesis via N-cadherin and β-catenin.^32^^,^^33^ Notably, overexpression of Tbx5 in the chick hindlimb suppresses Tbx4 activity, potentially disrupting normal limb patterning.^79^ Given their structural similarities and overlapping expression domains, Tbx4 and Tbx5 may either independently regulate shared downstream targets or form heterodimers to modulate transcriptional activity.

In cetaceans, evolutionary loss of hindlimb-specific enhancer elements, such as Hoxd10, Hoxc10, and Pitx1 binding sites within the HLEA region, has been proposed to underlie the degeneration of hindlimb structures, including skeletal, muscular, and neural components.^100^ These modifications are thought to result in altered Tbx4 expression patterns, reflecting the adaptive regression of hindlimbs in aquatic environments.

In conclusion, *TBX4* plays a pivotal role in hindlimb development through its interactions with multiple transcription factors and signaling pathways, including FGF, Wnt, and RA. These interplays are crucial for limb outgrowth, skeletal formation, and soft tissue morphogenesis. The dysregulation of *TBX4* has been implicated in various human limb disorders such as SPS, DDH, and clubfoot. Additionally, evolutionary changes in *TBX4* regulatory elements contribute to the adaptation of limb structures in different species. Understanding these complex mechanisms provides valuable insights into vertebrate limb development and its associated pathologies.

**Figure 4:**
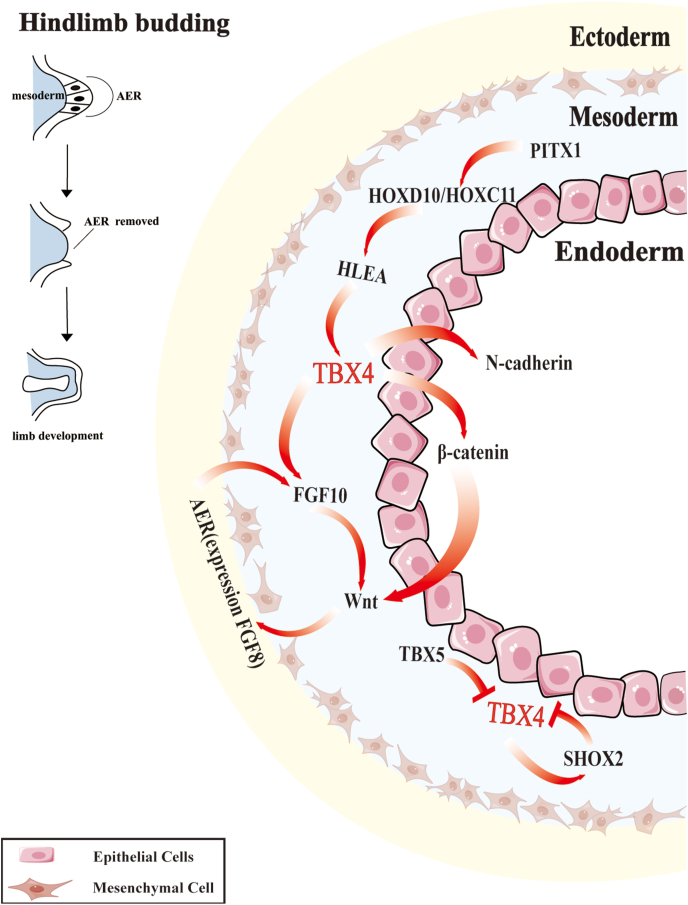
TBX4-mediated regulatory network in limb development. This figure illustrates that *TBX4* is crucial for mammalian limb development during the embryonic stage. It activates *FGF10* within the mesoderm and interacts with β-catenin in the Wnt pathway, driving cell proliferation and differentiation. N-cadherin, a cell adhesion molecule, regulates cell interactions, stabilizing the Tbx4-active cellular environment and maintaining limb structure integrity. HLEA may participate in early limb bud formation, collaborating with *Tbx4* to promote cell cycle progression and differentiation. Transcription factors like *Shox2*, *Hoxd10*, and *Hoxc11* interact with *Tbx4* to regulate limb growth and anteroposterior patterning. The apical ectodermal ridge (AER) secretes factors such as Fgf8, which interacts with the Tbx4-regulated Fgf10 pathway, sustaining continuous limb growth. Overall, these interconnected signaling pathways form a complex network, with Tbx4 serving as a key node ensuring orderly limb development from bud initiation to maturation.

To visualize the complex interplay of TBX4 with other key factors in hindlimb development, please refer to Figure 4, which depicts the intricate signaling network involving TBX4, PITX1, FGF10, and other regulatory elements that contribute to the morphological development of the hindlimb.

### TBX4’s role in cancer and its associated mechanisms

Accumulating evidence indicates that TBX4 expression is significantly associated with tumor grade, prognosis, and overall survival across a variety of cancers. In malignancies such as thyroid cancer,^101^^,^^102^ breast cancer,^103^ pancreatic cancer,^21^ certain non-small cell lung cancers,^104^ and liver cancer,^105^ *TBX4* may exert potential tumor-suppressive effects.

### Pancreatic ductal adenocarcinoma

In stage II pancreatic ductal adenocarcinoma, low TBX4 expression is generally correlated with poor prognosis.^21^^,^^104^ Interestingly, in a Wilms tumor patient harboring a WT1 mutation, pulmonary metastases displayed elevated TBX4 expression following six months of chemoradiotherapy, implying a potential role for *TBX4* in regulating tumor metastasis and therapeutic response. This finding also suggests that high TBX4 expression may be linked to improved prognosis in certain contexts.^106^ Further research in pancreatic ductal adenocarcinoma has shown that TBX4 expression decreases progressively with declining tumor differentiation, raising the possibility that reduced *TBX4* levels may serve as an indicator of increasing tumor aggressiveness. However, since the experimental cells may have originated from different lineages, the accuracy of this conclusion remains uncertain.^107^

### Intrahepatic cholangiocarcinoma

In intrahepatic cholangiocarcinoma, which shares morphological and biological features with pancreatic ductal adenocarcinoma,^21^^,^^105^ tumors with high TBX4 expression were of lower grade, whereas those with low TBX4 expression tended to be high-grade. Moreover, *TBX4* has been identified as a potential biomarker for disease progression in bladder cancer, with its methylation status linked to tumor advancement, though specific details are yet to be clarified.^108^

### Lung cancer

Even though in certain subsets of patients with non-small cell lung cancer, high TBX4 expression has been associated with poor prognosis. This phenomenon may be due to reduced expression of the lncRNA TTTY15 (short for testis-expressed transcript, Y-linked 15), which under normal conditions suppresses TBX4 via DNA methyltransferase 3 alpha (DNMT3A)-mediated DNA methylation.^109^ But in most patients with non-small cell lung cancer, low TBX4 expression is largely reported to be associated with poor prognosis.

From an epigenetic perspective, TBX4 expression is tightly regulated by DNA methylation, and its methylation status has emerged as a prognostic indicator in multiple cancer types. Aberrant DNA methylation, particularly in early-stage cancer, frequently occurs and influences gene expression.^110^^,^^111^ In lung cancer, TBX4 exhibits a hypermethylated state,^112^ and in lung adenocarcinoma, specific TBX4 methylation patterns are associated with pathogenesis and tumor-suppressive effects. Studies suggest that the TBX4-specific methylation marker cg14823851 demonstrates high sensitivity and specificity for distinguishing lung adenocarcinoma from squamous cell carcinoma.^113^^,^^114^ Notably, TBX4 may also function through other mechanisms in cancer. For instance, in both pancreatic ductal adenocarcinoma and normal pancreatic tissues, the TBX4 promoter region displays high CpG island methylation, suggesting that additional factors regulate TBX4 expression in the pancreas.^21^ In adenoid cystic carcinoma tissues, studies have also found that the CpG island near the TBX4 gene showed a hypermethylation state.^115^ Furthermore, cytoplasmic TBX4 expression in pancreatic cancer cells is linked to nuclear export signal-mediated protein shuttling, though the precise mechanism remains unclear.^116^^,^^117^

Collectively, these findings highlight the complex role of TBX4 in cancer initiation, progression, and metastasis, with its expression levels and methylation status emerging as potential biomarkers for cancer diagnosis and therapy.

### TBX4 in other systems and associated mechanisms

In addition to its well-established role in human development, TBX4 has also been implicated in a variety of pathological conditions. Emerging evidence suggests that TBX4 may not only associate with cleft lip/palate and isolated cleft palate but also interact with maternal folate intake, although this relationship warrants further validation.^22^^,^^118^ Notably, many studies identified TBX4 haploinsufficiency as a potential causative factor not only for the previously mentioned developmental delay and limb malformations, but also, interestingly, for microcephaly and cardiac anomalies.^22^^,^^119^^,^^120^ Functional analyses by Infante et al (2015) demonstrated that the TBX4 enhancer, hindlimb enhancer B (HLEB), regulated vulvar development in both mice and snakes, underscoring its evolutionary conservation.^121^ Embryonic lethality studies demonstrated that TBX4-null mouse embryos exhibited reduced primordial germ cell populations or complete depletion, suggesting potential long-term fertility implications in adulthood.^122^ Experimental evidence from Li et al (2019) showed TBX4 down-regulation in ethylene thiourea-induced anorectal malformations in rat embryos.^123^ Mechanistically, Yu et al (2021) uncovered that TBX4 modulated the expression of E74-like ETS transcription factor 4 (ELF4), RUNX family transcription factor 3 (RUNX3), and zinc finger MIZ-type containing 1 (ZMIZ1) in B cells of systemic lupus erythematosus patients, contributing to systemic lupus erythematosus immunopathology.^124^ Concurrently, emerging research revealed that ocu-miR-29-5p indirectly inhibited *Pasteurella multocida* invasion by regulating host immune response genes, including TBX4.^125^ Collectively, these findings provide novel insights into the multifaceted roles of TBX4 in disease pathogenesis and highlight critical knowledge gaps, particularly regarding how TBX4 mutations influence disease severity and clinical heterogeneity, an area with substantial translational research potential.

## Conclusions and perspectives

Research on *TBX4* has unveiled significant therapeutic potential across a range of diseases, from pulmonary fibrosis to PAH, and from tracheal cartilage development to engineered cartilage reconstruction.

In the context of pulmonary fibrosis treatment, emerging studies suggest that inhibiting collagen prolyl hydroxylase may reduce excessive collagen deposition, potentially slowing down or even preventing the progression of lung tissue fibrosis, offering a new strategy for treatment.^126^ As a critical transcriptional factor, *TBX4* regulates the fibroblast invasiveness, the key contributor to fibrosis. Targeting *TBX4* and its downstream factors could modulate fibroblast activity and function across multiple pathways, offering a promising intervention for pathogenic pulmonary fibrosis progression.^34^ Research has also begun to elucidate TBX4’s role in PAH,^17^ indicating that precise diagnostics and management strategies may soon be applicable.

Additionally, TBX4’s involvement in tracheal cartilage development and engineered cartilage reconstruction holds potential for advancing tissue repair and regenerative engineering with scalable and biomechanical capabilities.^41^^,^^127^ In summary, the growing body of evidence highlights the significant role of TBX4 in disease mechanisms and underscores the need for further research to fully explore its therapeutic potential. Continued investigation into TBX4’s functions could lead to novel strategies for treating a variety of conditions.

Future research on TBX4 should include the application of heterozygous knockout mouse models and inducible knockout systems targeting TBX4 in specific organs at precise time points. Combining cell-type-specific investigations of *TBX4* mutations with drug screening and signaling pathway analyses could elucidate its functions across different cell types and tissues, as well as its mechanistic roles in disease,^101^ enhancing our understanding of embryonic development and organogenesis.

In the fields of stem cell therapy and regenerative medicine, the influence of TBX4 on tissue repair and regeneration holds promise for more effective treatments, particularly for pulmonary and orthopedic conditions. Furthermore, in-depth studies of TBX4 could benefit research on rare genetic diseases, such as SPS, PAH, and clubfoot.^10^^,^^57^^,^^98^ Expanding the cohort of individuals affected by TBX4-related diseases, combined with clinical assessments, will accelerate the understanding of these rare disorders, ultimately improving clinical diagnosis and treatment options.

Strengthening international collaboration and establishing global databases will be crucial in advancing TBX4-related research, facilitating its clinical translation, and driving medical innovations. Continued exploration of TBX4 is poised to revolutionize medicine, offering novel approaches for disease treatment and steering medical practice toward more personalized and precise therapies, ultimately providing patients with a broader array of treatment options and improving overall outcomes.

## CRediT authorship contribution statement

**Lin Yi:** Conceptualization, Visualization, Writing – original draft. **Li Zhou:** Conceptualization, Writing – original draft, Writing – review & editing. **Bianfei Shao:** Conceptualization, Formal analysis, Visualization. **Tingxiu Xiang:** Writing – review & editing, Funding acquisition, Investigation, Project administration. **Jingyi Tang:** Visualization, Validation, Writing – review & editing, Conceptualization.

## Data availability

All the data and materials supporting the conclusions were included in the main paper.

## Funding

This study was supported by the 10.13039/501100001809National Natural Science Foundation of China (No. 82172619) and the 10.13039/501100005230Natural Science Foundation of Chongqing, China (No. CSTC2021jscx-gksb-N0023).

## Conflict of interests

Tingxiu Xiang is the Associate Editor of *Genes & Diseases*, but he/she has no involvement in the peer-review of this article and has no access to information regarding its peer-review. The rest authors declared no conflict of interests.
